# OGDHL Is a Modifier of AKT-Dependent Signaling and NF-κB Function

**DOI:** 10.1371/journal.pone.0048770

**Published:** 2012-11-12

**Authors:** Tanusree Sen, Nilkantha Sen, Maartje G. Noordhuis, Rajani Ravi, T-C Wu, Patrick K. Ha, David Sidransky, Mohammad Obaidul Hoque

**Affiliations:** 1 Department of Otolaryngology-Head and Neck Surgery, Johns Hopkins University School of Medicine, Baltimore, Maryland, United States of America; 2 Solomon H. Snyder Department of Neuroscience, Johns Hopkins University School of Medicine, Baltimore, Maryland, United States of America; 3 Department of Pathology, Johns Hopkins Medical Institutions, Baltimore, Maryland, United States of America; 4 Milton J Dance Jr. Head and Neck Center, Greater Baltimore Medical Center, Baltimore, Maryland, United States of America; Sun Yat-sen University Medical School, China

## Abstract

Oxoglutarate dehydrogenase (OGDH) is the first and rate-limiting component of the multi-enzyme OGDH complex (OGDHC) whose malfunction is associated with neuro-degeneration. The essential role of this complex is in the degradation of glucose and glutamate and the *OGDHL* gene (one component of OGDHC) is down-regulated by promoter hypermethylation in many different cancer types. These properties suggest a potential growth modulating role of OGDHL in cancer; however, the molecular mechanism through which OGDHL exerts its growth modulating function has not been elucidated.

Here, we report that restoration of OGDHL expression in cervical cancer cells lacking endogenous OGDHL expression suppressed cell proliferation, invasion and soft agar colony formation *in vitro*. Knockdown of OGDHL expression in cervical cancer cells expressing endogenous OGDHL had the opposite effect. Forced expression of OGDHL increased the production of reactive oxygen species (ROS) leading to apoptosis through caspase 3 mediated down-regulation of the AKT signaling cascade and decreased NF-κB phosphorylation. Conversely, silencing OGDHL stimulated the signaling pathway via increased AKT phosphorylation. Moreover, the addition of caspase 3 or ROS inhibitors in the presence of OGDHL increased AKT signaling and cervical cancer cell proliferation.

Taken together, these data suggest that inactivation of OGDHL can contribute to cervical tumorigenesis via activation of the AKT signaling pathway and thus support it as an important anti-proliferative gene in cervical cancer.

## Introduction

Cervical Cancer is the second most common cancer among women worldwide, responsible for about 500 000 new cases and 250 000 deaths each year (http://www.who.int/reproductivehealth/topics/cancers/en/). Most cases and deaths occur in developing countries, whereas in developed countries, the implementation of population-based screening programs (based on cytomorphological examination of cervical smears) for cervical cancer and its precursor lesions (cervical intraepithelial neoplasia), has caused a significant reduction in cervical cancer incidence blunted.

Depending on the stage of the disease at presentation, cervical cancer patients are treated surgically (early stage) or with chemo-radiation (locally advanced stage). Although the addition of chemotherapy to standard radiotherapy has improved survival of cervical cancer, the 5-year survival rate of patients treated with chemoradiation is only 66% [2]. Further improvement of survival rates by intensification of standard chemoradiation is limited, because of resistance to radiotherapy and/or chemotherapy and an increase of short- and long-term side effects [3]. Therefore, new treatment modalities are urgently needed to increase the anti-tumor effect of chemo-radiation and thereby improve the survival of cervical cancer patients. For the development of novel personalized treatment modalities, it is essential to understand cervical cancer at a molecular level. The molecular carcinogenesis of cervical cancer is characterized by multiple alterations of gene expression and function. These changes arise from a series of molecular and morphological events affecting oncogenes and tumor suppressor genes (TSGs), all leading inexorably to abnormal changes in cell signaling transduction pathways.

OGDHL is one of the rate-limiting component of the multi-enzyme OGDH complex (OGDHC) whose malfunction is associated with neuro-degeneration [4]. We have previously shown that the promoter of *OGDHL* is differentially methylated in different tissue types, and cancer-specific promoter methylation was also observed in breast, cervix, lung, oesophagus, pancreas and colon cancers, while methylation was absent in ovary, kidney and bladder cancers [5], [6]. Furthermore, altering the promoter methylation by treatment with a demethylating agent (5-Azacytidine) in cervical cancer cell lines led to re-expression of OGDHL [5]. Epigenetic alteration is now considered as one of the hallmarks of cancer and cancer specific promoter hypermethylation occurred in tumor suppressor genes. Although epigenetic study findings suggest OGDHL as a potential cancer related gene, the direct genetic evidence of growth modulating function of OGDHL is lacking. In the present study we sought to elucidate the functional aspects of OGDHL inactivation in cervical tumorigenesis.

## Materials and Methods

### Chemicals and antibodies

DMSO, N-acetyl l-cysteine or NAC, butylated hydroxytoluene or BHT, DEVD-FMK and antibodies against β-actin and flag were obtained from Sigma Chemical Co. (St. Louis, MO). DMEM, fetal bovine serum (FBS), Lipofectamine RNAiMAX reagent, TRIzolTM reagent, and PCR primers for OGDHL and GAPDH were purchased from Invitrogen Corporation (Carlsbad, CA). FuGENE HD transfection reagent was from Roche Molecular Biochemicals (Indianapolis, IN). The Caspase 3 Assay kit was obtained from Calbiochem (San Diego, CA). The BrdU cell proliferation kit was purchased from Roche Applied Science (Indianapolis, IN). 8.0 µm PET Membrane 24-well Cell Culture Matrigel Invasion Chambers were purchased from BD Biosciences (San Jose, CA). Ready-to-use in BD Falcon™ TiterTACS™ in situ Apoptosis Detection Kit was obtained from R&D System Inc. (Minneapolis, MN). NF-κB, Secreted Luciferase Reporter System was purchased from Clonetech Laboratories Inc., (Palo Alto, CA). Mouse anti-human cytochrome C, PARP, NF-κB, Cox–II and the horseradish peroxidase (HRP)-conjugated anti-rabbit IgG and anti-mouse IgG were obtained from Santa Cruz Biotechnology (Santa Cruz, CA), rabbit anti-human total AKT, phospho-NF-κB (S536), phospho-PI3K [p85 (Tyr458)/p55 (Tyr199)] and phospho-AKT (S473), lamin and caspase 3 antibodies were obtained from Cell Signaling Technology (Beverly, MA), anti-human OGDHL antibodies are from Abcam (ab100928, Cambridge, MA ) and ProteinTech Group, Inc (Chicago, IL).

### Semi-quantitative RT-PCR

Total RNA was isolated from cells using the RNeasy Kit (Qiagen) according to manufacturer's instructions. First-strand cDNA was synthesized from 1 µg of total RNA using qScript™ cDNA SuperMix kit (Quanta Biosciences, Gaithersburg, MD). Each RT-PCR reaction consisted of 25 or 30 cycles of 1 min at 94°C, 1 min at 55°C and 1 min at 72°C. Quantitation of the amount of PCR product was performed after electrophoresis on 1% agarose gels and ethidium bromide staining. Primers used for amplification of OGDHL and GAPDH (internal controls) are as follows: OGDHL F: TCGAGGTGAGCCAGCTCTAT, R: GCACCTGGGTACTTCTCTGC; GAPDH F: GAGTCAACGGATTTGGTCGT, R: TTGATTTTGGAGGGATCTCG. The PCR product was confirmed by direct sequencing.

### Cell Culture, transfection and subcellular protein isolation

Human cervical cancer cell lines SiHa, CaSki, HeLa and ME180 were obtained from American Type Culture Collection (ATCC) and maintained in DMEM (high glucose) complete medium supplemented with 2 mM L-glutamine, 10% FBS and 1% penicillin-streptomycin. All cells were cultured in a humid atmosphere at 37°C with 5% CO_2_. Cells were transiently transfected with the control expression vector or expression vector containing Flag-OGDHL, HA-AKT1, or EGFP-MnSOD using FuGENE HD transfection reagent in accordance with the manufacturer's specifications. We used ON-TARGET*plus* siRNA for OGDHL (Dharmacon, FLJ10851) that consist of four individual siRNAs targeting a single gene OGDHL for greater target specificity when down-regulating OGDHL. For confirming specificity, cells were transfected with a scrambled siRNA (ON-TARGET*plus* Non-targeting Pool, D-001810-10-05) at a total concentration of 20 nM. Silencing of OGDHL was examined 48–72 h after transfection. Human AKT1 siRNA was obtained from Cell Signaling Technology. Cells were transfected with 20 nM siRNA using the Lipofectamine RNAiMAX reagent. Cells were harvested after 24, 48 and 72 hours post transfection as per the assay requirement. Nuclear and cytoplasmic fractions were prepared as described previously [7].

### Plasmid constructs

OGDHL-Flag was obtained commercially from Origene (Rockville, MD) in a PCMV6-Entry vector. Hemagglutinin (HA) tagged AKT1 clone (pcDNA3 Myr HA AKT1) and pBI-EGFP-MnSOD vectors were purchased from Addgene (Cambridge, MA).

### Measurement of reactive oxygen species

The production of reactive oxygen species (ROS) from intact cells was measured using H_2_DCF-DA as published earlier [8]. H_2_DCF-DA is a non-polar compound and is hydrolyzed within the mitochondria to form a non-fluorescent derivative, which, in the presence of a proper oxidant (H_2_O_2_ and possibly other reactive oxygen species), is converted to a fluorescent product. Briefly, the cells were incubated with H_2_DCF-DA (10 µM) in PBS at 37°C for 30 min. At the end of the incubation cells were washed with PBS and resuspended in 1 ml of the same buffer, and the fluorescence was measured spectrofluorometerically (*λ*_ex_ 507 nm, *λ*_em_ 530 nm).

### Estimation of fluorescent lipid peroxidation products

The steady-state level of peroxidative damage markers in whole cell lysate was assessed by estimating fluorescent lipid peroxidation products after solubilizing the cell pellet in 2 ml of 15% sodium-dodecyl-sulphate in phosphate buffered saline solution, pH 7.4. The fluorescence intensity indicating the total fluorescent lipid peroxidation products were measured at excitation 360 nm and emission 430 nm [8].

### Immunoblotting analysis

Cells were lysed on ice for 30 min in RIPA buffer [150 mM NaCl, 100 mM Tris (pH 8.0), 1% Triton X-100, 1% deoxycholic acid, 0.1% SDS, 5 mM EDTA, and 10 mM NaF, supplemented with 1 mM phenylmethylsulfonylfluoride and protease inhibitor mixture (Sigma Chemical Co)]. After centrifugation at 12,000 rpm for 15 min, the supernatant was harvested as the total cellular protein extract. The protein concentration was determined using a Lowry protein assay (Bio-Rad Laboratories). Equal amounts of protein (30 µg each) were mixed with Laemmli (Bio-Rad Laboratories) sample buffer (62.5 mM Tris-HCl, pH 6.8, 2% SDS, 10% glycerol, 0.1 M DTT and 0.01% bromophenol blue), run on 4–12% NuPAGE, and electroblotted onto Nitrocellulose membrane (Bio-Rad Laboratories, Richmond, CA). The membrane was blocked with phosphate buffer saline (PBS) supplemented with 0.1% Tween 20 and 5% nonfat milk or 5% BSA for 1 h at room temperature, and probed with primary antibody overnight at 4°C followed by HRP-conjugated appropriate secondary antibody for 1 h at room temperature. Signals from immunoreactive bands were detected by enhanced chemiluminescence reagent (Santa Cruz Biotechnology).

### Immunofluorescence studies

Immunofluorescence was performed on ME180 cells as described previously [9], [10]. The OGDHL primary polyclonal rabbit antibody from ProteinTech Group Inc. (Chicago, IL) was used at a concentration of 1∶300. Goat anti-rabbit IgG conjugated with Cy3 was used as a secondary antibody at a concentration of 1∶1000 (Jackson Immunoresearch, West Grove, PA). Cells were co-loaded with MitoTracker Green FM (200 nM, 37°C, and 30 min). Experiments were carried out by using a simplified Ca^2+^-free Hepes-buffered medium (HSS) whose composition was: 120 mM NaCl/5.4 mM KCl/0.8 mM MgCl_2_/20 mM Hepes/15 mM glucose/10 mM NaOH, pH 7.4. Confocal images were obtained by using Zeiss confocal microscope equipped with the LSM 510 attachment using a 20× objectives, and argon (Ex: 488 nm; Em: 510 nm, for MitoTracker Green) and krypton (Ex: 550 nm; Em: 570 nm, for OGDHL) lasers.

### Cell viability and proliferation assays

Cellular viability was measured by the 3-(4, 5-dimethyl thiazol-2-yl)-2, 5-diphenyl tetrazolium bromide (MTT) proliferation assay kit (ATCC) according to the manufacturer's instructions. Briefly, 1×10^4^ cells were seeded in 96-well culture plates and cultured in 5% FBS for 24 h. 10 µl of MTT labeling reagent (5 mg/ml MTT) was added to the culture media without FBS, which was then incubated in the dark for a further 4 hours at 37°C. This step was followed by cell lysis with the addition of 1 ml of an SDS-based detergent reagent from the MTT kit. The plates were incubated for 2 h at 37°C to dissolve formazan crystals. Spectrophotometric readings (A_570 nm_-A_650 nm_) were obtained on a Spectra Max 250 96-well plate reader (Molecular Devices, Sunnyvale, CA). Each assay was carried out in triplicate and each experiment was repeated at least three times. Data are represented as the extent of cellular survival expressed as a percentage of control.

Cell proliferation was also determined by the incorporation of BrdU into cell nuclei using commercially available kit (Roche Molecular Biochemicals). Briefly cells were seeded in a 96-well culture plate at a density of 2.0×10^3^ cells per well. Forty-eight hours after transfection, cells were labeled for 2 h with BrdU. BrdU incorporated into cellular DNA was quantified as instructed by the manufacturer.

### Terminal deoxynucleotidyl transferase dUTP nick end labeling (TUNEL) assay

Following the manufacturer's instructions, DNA damage was evaluated and quantified with a colorimetric apoptosis detection kit (Titer TACS; R&D System, Minneapolis, MN) that uses TUNEL staining in a 96-well format. Briefly, cells were cultured and transfected in 12-well plates. 48 hours after transfection, cells were trypsinized and suspended in DMEM; the cells were then counted and transferred into a round-bottom 96-well plate (2×10^5^cells/well). Cells were then fixed with 3.7% buffered formaldehyde for 5 min followed by washing with PBS. Next, the cells were permeabilized with 100% methanol for 20 min followed by washing with PBS. Cells were then subjected to labeling procedure following the manufacturer's instructions. The reaction was stopped with 2 N HCl after 30 minutes of substrate addition and the absorbance was measured at 450 nm with microplate reader. For comparison, a positive control (nuclease treated control) was kept to confirm the permeabilization and labeling reaction.

### Caspase 3 cleavage assay

Caspase3 activity was measured using the Colorimetric Caspase-3 Assay Kit (Calbiochem) following manufacturer's instruction. Briefly, at the end of the exposure; cells (1×10^6^ cells) were harvested, counted and washed twice with ice-cold PBS and then lysed in 50 µl of lysis buffer [50 mM HEPES, pH 7.4, 100 mM NaCl, 0.1% 3(3-cholaminopropyl diethylammonio)-1-propane sulfonate, CHAPS, 5 mM dithiothreitol (DTT), 0.1 mM ethylenediamine tetraacetic acid (EDTA)], incubated for 5 min on ice, and then centrifuged at 10,000×*g* for 10 min at 4°C. Ten-microliters of the supernatants were incubated with 200 µM Ac-DEVD-*p*NA substrate at 37°C in the presence or absence of 0.1 µM caspase-3 inhibitor (Ac-DEVD-CHO). The reaction was stopped in addition of hydrochloric acid (1 N) and the absorbance (405 nm) was determined after 3 h incubation at 37°C. A sample with protein and buffer without colorimetric substrate was taken as control or basal value. The relative increase in caspase 3 activity was determined by comparing the levels with the untreated control.

### *In Vitro* Caspase Inhibition

To assess the role of caspases in OGDHL-induced AKT cleavage, cells were pre-incubated for 2 h with caspase 3 inhibitor DEVD-FMK (25 µm) in serum-free medium and subsequently transfected with OGDHL for 48 h.

### Caspase 3 mediated AKT Cleavage Assay

Cell lysate (50 µg of total protein) was incubated for 0, 2, 4, or 6 h at 37°C in PIPES assay buffer [PIPES (20 mM), NaCl (100 mM), DTT (10 mM), EDTA (1 mM), 3-[(3-cholamindopropyl)-dimethylammonio]-1-propane-sulfonic acid (0.1%; w/v), and sucrose (10%; w/v); pH 7.2] containing 10 µg of recombinant active caspase 3 in the absence or presence of caspase-inhibitor DEVD (25 µm). Total volume of the reaction was 40 µl. The incubation was terminated with the addition of an equal volume of 2×sample buffer [Tris-HCl (100 mM; pH 6.8), DTT (200 mM), SDS (4%; w/v), glycerol (20%; v/v), and bromphenol blue (0.2%)]. AKT cleavage was then assessed by Western blotting.

### Anchorage-independent colony formation analysis in soft agar

Anchorage-independent cell growth was analyzed by plating 0.36% top agarose (Bacto Agar: Becton, Dickinson, Sparks, MD) containing 1×10^5^ cells (stable clone constitutively expressing OGDHL and empty vector as control) mixed with corresponding medium (DMEM, high glucose) with 10% fetal bovine on a surface of 0.72% bottom agarose mixed with DMEM with 10% FBS in 6-well plates at 37°C. Cells were fed weekly by overlaying with fresh soft agar solution, and colonies were photographed and counted under a light microscope after 3 weeks of incubation. Each experiment was performed in triplicate wells and repeated 3 times.

### Invasion assay

Matrigel invasion assay was performed with SiHa and HeLa cells after forced expression and knockdown of OGDHL in respective cell line. BioCoat Matrigel (BD Biosciences, Bedford, MA) that reconstitutes the basal membrane was purchased to assess cell invasion. 24-well tissue culture plate inserts coated with Matrigel were re-hydrated for 2 h in 37°C with media containing 5% FBS. Media (0.6 ml) containing 5% FBS was added to each plate well as a chemo-attractant, and 0.2 ml (2×10^4^ cells) of cell suspension was added to each insert. After incubation for 24 h, non-invading cells were removed from the upper side of the membrane by scrubbing. Invasion of cells to the underside of the Matrigel-coated membrane was detected by staining the cells with crystal violet solution and visualizing the cells under a microscope. After staining, cells were counted under a microscope in four randomly selected fields (magnification×100), and results were expressed in the form of a bar graph. Assays were performed three times for each condition.

### EMSA of NF-κB DNA binding activity

Nuclear extracts were prepared using NE-PER buffer (Pierce, Rockford, IL). NF-κB electrophoretic mobility shift assay (EMSA) was performed according to the gel shift TM NF-κB p65 kit (Active Motif) protocol. Briefly, double-stranded oligonucleotides containing a consensus binding site for Rel-A were 5′ end-labeled using polynucleotide kinase and [^32^P] dATP. Nuclear extracts (2.5–5 µg) were incubated with ≈1 µl of labeled p65 Rel A oligonucleotide (20,000 c.p.m.) in a binding buffer provided by gel shift TM NF-κB p65 kit for 20 min at 4°C. The DNA-protein complexes were resolved on a 5% non-denaturing polyacrylamide gel and visualized by autoradiography.

### NF-κB luciferase assay

Cells were transiently transfected with OGDHL-flag or HA-AKT1 or control vector using FuGENE HD (according to the manufacturer's protocol) in the presence or absence of either the pNF-κB-MetLuc2-Reporter vector or pMetLuc2-Control vector and after 24 to 48 h of transfection the NF-κB, promoter activity was determined according to the manufacturer's protocol (Ready-To-Glow p NF-κB, MetLuc luciferase reporter system, Clonetech). Each transfection was performed in duplicate, and all experiments were repeated at least three times.

### Development of OGDHL expressing stable clones in SiHa cell line

For the transfection of SiHa cells, we used the mammalian expression vector (Origene, Rockville, MD). This vector contains a cytomegalovirus promoter allowing efficient transcription of the recombinant OGDHL-cDNA, and the neomycin resistance gene for easy selection of the transfected cells. Transfection of full-length OGDHL-cDNA and empty vector was performed by FuGENE HD transfection reagent according to the manufacturer's instructions. Selection was performed via the addition of 1000 µg/ml (Clonetech Laboratories Inc.) of G418 at final concentration. To detect the expression of OGDHL, stable transfectants were lysed in the sample buffer containing 200 µl of RIPA buffer (50 mM Tris-HCl, pH 7.5, 150 mM NaCl, 1% Nonidet P-40, 0.5% sodium-deoxycholate, 0.1% SDS) and 1 mM PMSF. Equal amounts of protein were separated by sodium dodecyl sulfate-polyacrylamide gel electrophoresis and subjected to western blotting using anti-mouse Flag antibody M2 (Sigma Chemical Co.) at a 1∶5000 dilution. Twenty positive clones and three control clones were picked after 21 days selection. Among these, three positive clones and one control clone (empty vector) were selected for further functional experiments.

### Statistical analysis

The data represent mean ± SD from independent experiments done for three to five times. Statistical analysis was performed by Student's *t* test at a significance level of *P*<0.05 to <0.001.

## Results

### OGDHL expression in cervical cancer cell lines

**Figure 1:**
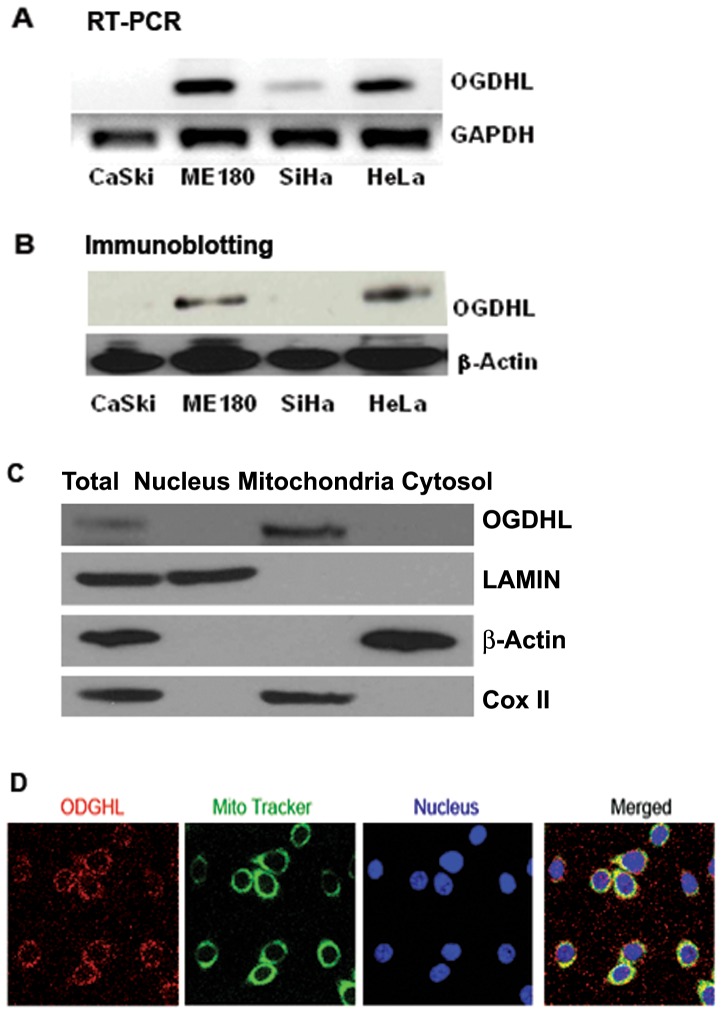
Expression and localization of OGDHL. **A.** mRNA expression of OGDHL in different cervical cancer cell lines. **B.** Protein expression of OGDHL in different cervical cancer cell lines **C.** OGDHL, β-actin, lamin and Cox-II in total, nuclear, mitochondria and cytosolic fractions of ME180 cell lines. **D.** Colocalization of OGDHL and MitoTracker Green in ME180 cell line. Cultures were co-loaded with the OGDHL (red fluorescence) and the mitochondrial marker, MitoTracker Green (green fluorescence), and imaged with confocal microscopy. Note the substantial overlap between OGDHL and MitiTacker (yellow), indicating that they largely target the same intracellular organelles.

We previously reported that OGDHL is down-regulated by promoter hypermethylation in cervical and breast cancer cell lines, and in a substantial portion of primary tumors of these two primary cancer types [1], [5], [6]. To understand the effect of deregulated OGDHL, we examined the mRNA expression of this gene in different cervical cancer cell lines and selected two cell lines (HeLa and ME180) that adequately expressed endogeneous OGDHL and two cell lines (SiHa and CaSki) that minimally expressed endogeneous OGDHL at mRNA and protein level (**Fig. 1A and B**). The present data were found to be consistent with our previous report, where we showed that the OGDHL gene was methylated and mRNA expression was silenced in the SiHa cell line [**5**], suggesting that expression of this gene may be regulated by promoter hypermethylation.

### Cellular localization of OGDHL

Earlier reports have established high sequence, structural, and functional similarity between OGDHL and OGDH [4]. OGDH is located exclusively within the mitochondria in tissues investigated, and there is no evidence for a cytosolic form of the enzyme [11]. Since OGDHL and OGDH are part of OGDHC (2-oxoglutarate dehydrogenase complex) and OGDH is completely localized in mitochondria, we attempted to determine the localization of OGDHL. As expected, subcellular fractionation showed that OGDHL was localized only in the mitochondrial fraction and not in the cytosolic or nuclear fractions (**Fig. 1C**) as determined by western blot. We next analyzed in detail the cellular staining patterns of OGDHL using fluorescence microscopy and observed a granular/tubular staining pattern reminiscent of mitochondria. As shown in **Fig. 1D**, when OGDHL co-loaded with the mitochondrial marker, MitoTracker Green, a high degree of co-localization was observed; the endogenous OGDHL overlapped with the mitotracker-positive-stained mitochondria in the cytoplasm. The antibody we used for western blotting was not suitable for immunofluorescence staining and we used the OGDHL antibody from another manufacturer (ProteinTech Group, Inc., Chicago, IL) for immunofluorescence study.

### Generation of oxidative stress and induction of apoptosis after OGDHL over-expression

**Figure 2:**
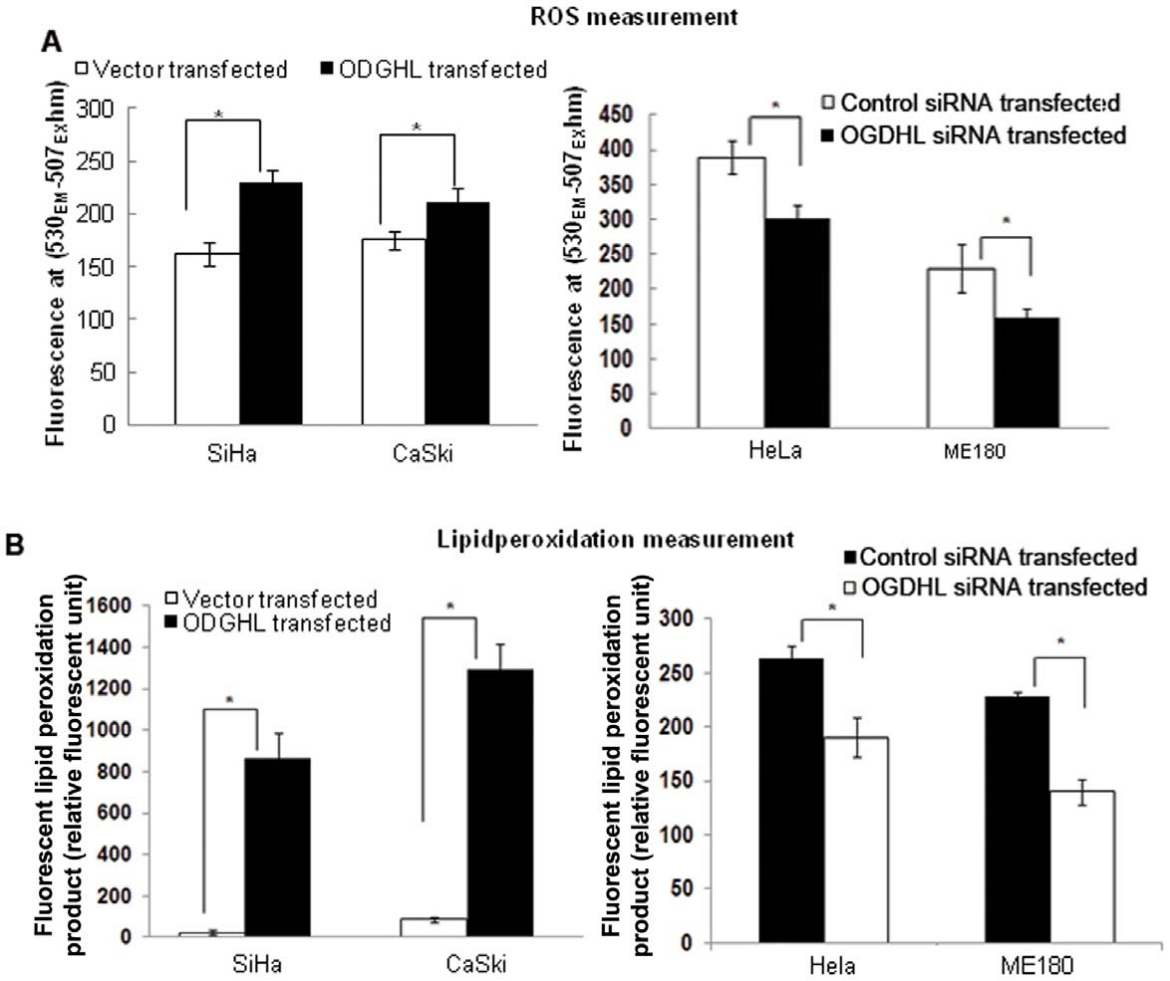
Modulation of ROS and Lipid peroxidation due to changes of OGDHL expression. **A.** ROS and **B.** Lipid peroxidation measurement in SiHa and CaSki cell lines after OGDHL over-expression (left panels) and in HeLa and ME180 cell lines after OGDHL siRNA mediated downregulation (right panels). OGDHL significantly increases the ROS and lipid peroxidation production in SiHa and CaSki Cell lines (*P<0.001 for each cell line for ROS and *P<0.05 for each cell line for lipid peroxidation). Similarly ROS and lipid peroxidation were decreased in HeLa and ME180 cell lines after knocking down of OGDHL (*P<0.001 for both cell lines for ROS and *P<0.05 for both cell lines for lipid peroxidation). OGDHL overexpression and down-regulation in respective cell lines was confirmed by western blotting (**Figure S 1**).

As mitochondria are considered to be the major intracellular source of Reactive Oxygen Species (ROS) and OGDHL is localized in mitochondria, we were interested to determine the level of oxidative stress by measuring ROS and lipid peroxidation in the cervical cancer cells after OGDHL overexpression or down-regulation. OGDHL was forcefully expressed in SiHa and CaSki and knocked down in HeLa and ME180 cell lines. We found significant increases in ROS production and lipid peroxidation (left panels) in SiHa and CaSki cell lines after OGDHL overexpression, compared to a significant decrease in normal ROS production and lipid peroxidation in HeLa and ME180 cell lines (right panels) after OGDHL knockdown (**Fig. 2A and 2B**). This observation supports the fact that OGDHL can significantly influence cellular ROS production and lipid peroxidation.

**Figure 3:**
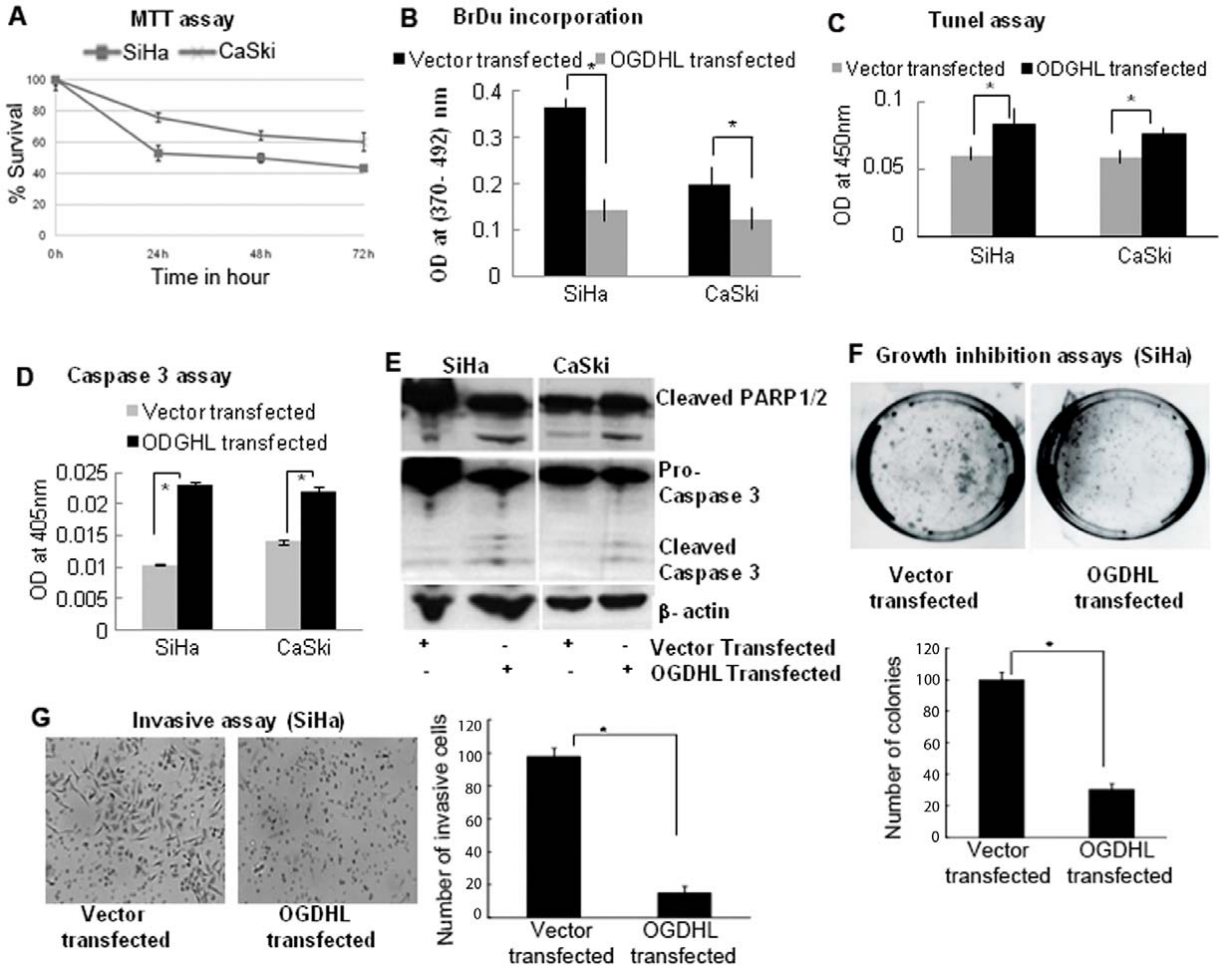
Phenotypic alterations of cells after forceful expression of OGDHL. **A.** **MTT assay**: Significantly decreased numbers of SiHa and CaSki cells were observed after forceful expression of OGDHL in a time dependent manner (after 0, 24, 48 and 72 h of transfection). Cell growth rate is expressed as absorbance at 570 to 650 nm (* ***P*** **<0.001**).; **B.** **BrdU assay**: OGDHL inhibit cell proliferation of SiHa and Caski cells as detected by DNA synthesis (*P<0.001 and 0.001 for SiHa and CaSki cells respectively); **C.** **Tunel assay**: Forty-eight hours after transfection of OGDHL, the ratio of apoptotic versus total number of cells was evaluated by TUNEL assay. Significant differences were observed between OGDHL and mock transfected cells (*P<0.001 and 0.001 for SiHa and CaSki cell lines respectively); **D.** **Caspase 3 assay**: Caspase-3 activity was measured using the Caspase colorimetric assay kit in SiHa and CaSki cells 48 hours after mock or OGDHL-transfection. (*P<0.001,**P<0.001 respectively); **E.** **Immunoblot analysis**: Cleaved Caspase 3 and PARP1/2 were observed in SiHa and CaSki cell lines after overexpression of OGDHL; **F.** **Colony formation assay**: The effect of exogenous OGDHL expression in colony formation of SiHa cell line. The SiHa cell line was transfected with constructs encoding OGDHL or the empty vector. Cells were harvested 24 h after transfection, and equal cell numbers were seeded in 100 mm dishes and grown under selection in G418 for 21 days. The representative photograph after 21 days (left) and the quantification of the number of G418 selected SiHa cell colonies counted in 3 plates (right). The vector control was set at 100%. The data represent the mean ±SD of three independent experiments, each done in triplicate. **G.** **Invasive assay**: SiHa cells 48 hours after transient transfection of OGDHL and empty vector. Cells that invaded the polycarbonate membrane of transwell chamber (Left). The number of cells that invaded the polycarbonate membrane of transwell chamber (right) (*P<0.001). The data represent the mean ±SD of three independent experiments, each done in triplicate.

**Figure 4:**
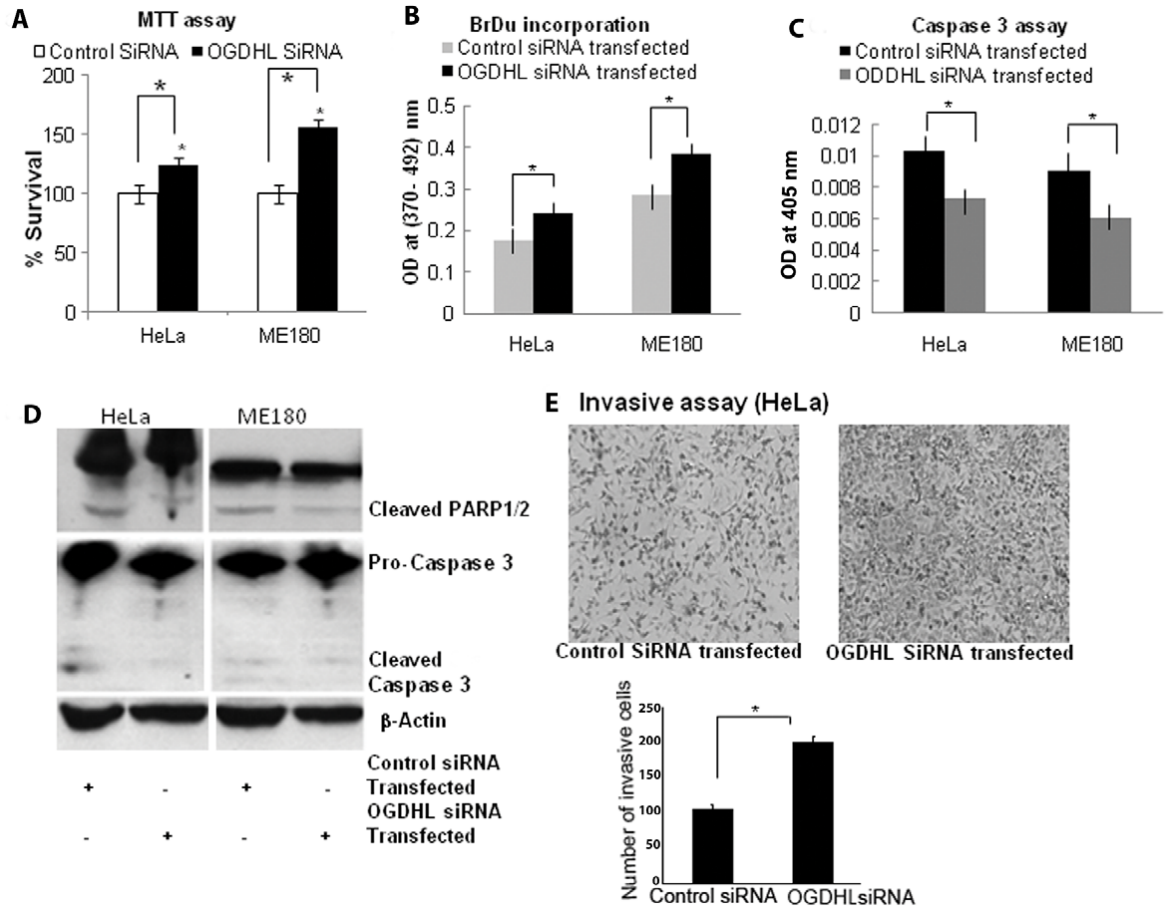
Phenotypic alterations of cells after siRNA mediated down-regulatin of OGDHL. **A.** **MTT assay:** MTT assay 48 hours after transient transfection of OGDHL siRNA in HeLa and ME180 cell lines; Cell growth rate is expressed as absorbance at 570 to 650 nm **B.** **BrdU assay:** OGDHL siRNA increased cell proliferation of HeLa and ME180 cells as detected by DNA synthesis (*P<0.001 and 0.001 for HeLa and ME180 cells respectively); **C.** **Caspase 3 assay:** Caspase-3 activities in HeLa and ME180 cells were measured in mock or 48 hours after OGDHL siRNA-transfected cells using the Caspase colorimetric assay kit. *P<0.001. **D.** **Immunoblot analysis**: Immunoblotting analysis of Caspase 3 and PARP1/2 in HeLa and ME180 cell lines after siRNA mediated knockdown of OGDHL and **E.** **Invasive assay**: Invasive assay with HeLa cells 48 hours after transient down-regulation of OGDHL by siRNA and scramble siRNA (control). Representative photograph of HeLa cells that invaded the polycarbonate membrane of transwell chamber (Left). The number of HeLa cells that invaded the polycarbonate membrane of transwell chamber were significantly more when OGDHL expression were inhibited by siRNA (* *P*<0.001) (right). The data represent the mean ±SD of three independent experiments, each done in triplicate.

It is known that Reactive Oxygen Species (ROS) contribute to neuronal and cancer cell death caused by different stimuli [10], [12], [13], [14]. Cell death can be prevented by blocking ROS or by removing ROS or ROS byproducts from the cell culture medium, suggesting that *OGDHL*-mediated ROS production may play a critical role in the process of apoptosis induction [5], [15]. Thus, having determined that *OGDHL* is frequently methylated, we examined its potential tumor suppressor function in cervical cancer cell lines. To evaluate the effect of OGDHL on the growth of cervical cell lines, OGDHL was forcefully expressed in SiHa and CaSki cell lines, and siRNA mediated knockdown of OGDHL was performed in HeLa and ME180 cell lines. Verification of OGDHL overexpression and down-regulation were done by RT-PCR and immunoblotting analysis of OGDHL 48 h after transfection (**Figure S1 A, B** respectively). Cell viability was determined by the MTT assay. As shown in **Fig. 3A**, forced expression of OGDHL significantly inhibited growth of SiHa and CaSki cell lines in comparison with vector transfected controls where cell growth inhibition is mediated in a time-dependent manner. To determine whether the decreased number of cells was due to cell death or decreased cell proliferation, we performed the BrdU assay which evaluates changes in DNA synthesis and is another indirect measurement for cell proliferation. We used OGDHL transfected and empty vector transfected cell lines, and **Fig. 3B** shows decreased cell numbers in OGDHL transfected cells, which indicates that the decrease cell number is partly related to decrease cellular proliferation. Moreover, the TUNEL assay, which detects DNA strand breaks by labeling free 3′-OH termini, showed that OGDHL-transfected cells were undergoing the early stages of apoptosis (**Fig. 3C**). Data from caspase-3 activity assay (**Fig. 3D**) and caspase 3 and poly-ADP-ribose polymerase (PARP) protein cleavage immunoblotting (**Fig. 3E**) analysis also indicated that OGDHL overexpression induced apoptosis in cervical cancer cells that lack expression of OGDHL. To assess long-term growth, colony focus assays were done after treatment of transfected cells with the plasmid selection marker, G418. OGDHL showed potent growth-suppressive activity by markedly reducing the colony-forming ability of the cells (**Fig. 3F**). In an invasion assay, we found that the number of invasive SiHa cells decreased after 48 h of OGDHL transfection, compared to that of empty vector transfected controls (**Fig. 3G**). An opposite effect was observed after knock-down of OGDHL in ME180 and HeLa cell lines as shown in **Fig. 4**.

To show a direct regulatory role of OGDHL on cell growth, apoptosis and invasion, we performed rescue experiments involving OGDHL. We over expressed OGDHL in siRNA knockdown phenotype of cervical cancer cell lines. Rescue of the observed knockdown phenotype demonstrated an original and more stringent validation of the siRNA's selectivity and the phenotype specificity for OGDHL. As shown in **Figure S2A**, down-regulation of OGDHL by siRNA significantly increased the growth of HeLa and ME180 cells in comparison with scramble siRNA transfected controls. Further, rescuing OGDHL expression in OGDHL knockdown cells showed significant decrease in cell growth compare to that of only OGDHL knockdown cells (**Figure S2A**). Data from caspase-3 activity assay (**Figure S2B**) and caspase 3 and poly-ADP-ribose polymerase (PARP) protein cleavage immunoblotting (**Figure S2C**) analysis also indicated that OGDHL knock-down increased cell growth. Re-expression of OGDHL in the same cells showed a significant increase in caspase-3 activity (**Figure S2B**) and caspase 3 and poly-ADP-ribose polymerase (PARP) protein cleavage compare to that of only OGDHL knockdown cells (**Figure S2C**). In an invasion assay, we found that the number of invasive HeLa cells increased after 48 h of OGDHL siRNA transfection, compared to that of scramble siRNA transfection as controls (**Fig. 3D and E**). As expected rescuing OGDHL expression in OGDHL knockdown cells showed significant decrease in invaded cells compare to that of only OGDHL knockdown cells (**Figure S3D** and **E**). Taken together, these data support the notion that *OGDHL* has anti-proliferative properties in cervical cancer cell lines.

### Alteration of AKT pathway after OGDHL over-expression or down-regulation

**Figure 5:**
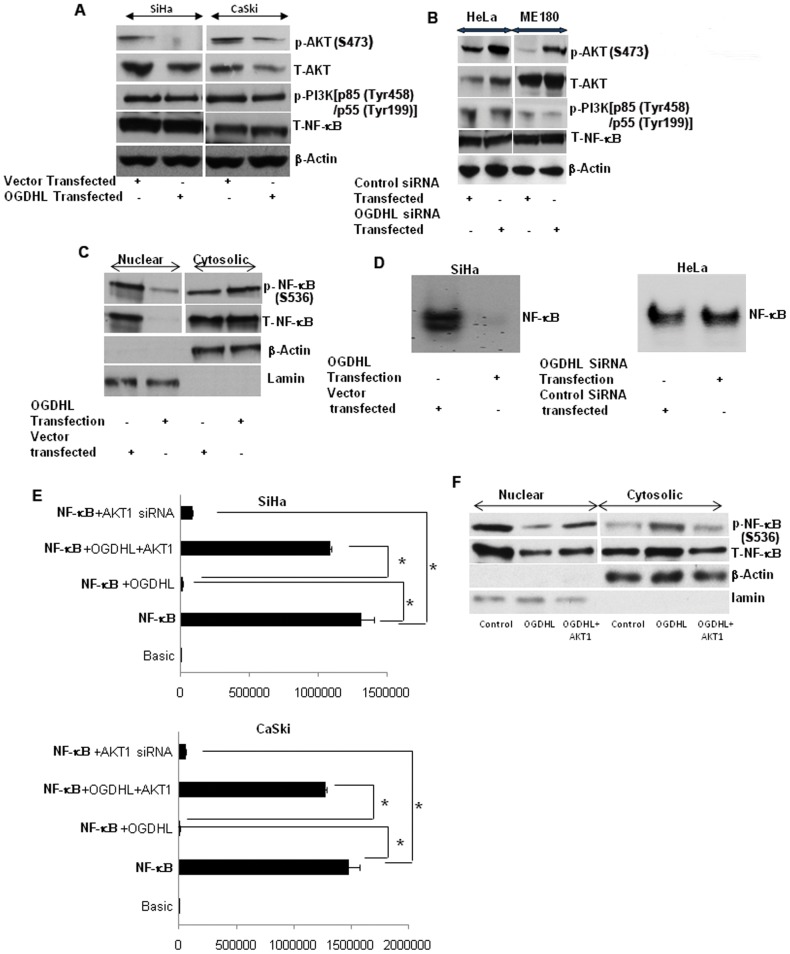
Immunoblotting analysis of phospo-AKT , total-AKT, phospo-PI3K, total- NF-κB and β-actin. In **A.** SiHa and CaSki cell lines 48 hours after transient transfection of OGDHL and empty vector; and **B.** HeLa and ME180 cell lines after OGDHL siRNA and scramble siRNA transfection; OGDHL inhibition by OGDHL siRNA has dramatic effect on total AKT and phospho-AKT level **C.** Immunoblotting analysis of phospo-NF-κB and total-NF-κB of SiHa cell lines 48 hours after OGDHL and empty vector transfection; β-actin and lamin was used as a loading control for cytosolic and nuclear fraction respectively; NF-κB translocation from cytoplasm towards the nucleus was decreased after OGDHL overexpression in SiHa cells; **D.** NF-κB gel shift assay in SiHa and HeLa cell lines; OGDHL overexpression in SiHa cells decreased the DNA binding activity of NF-κB compared to that of empty vector control; OGDHL siRNA increase the binding activity of NF-κB in HeLa cell; **E.** NF-κB luciferase assay in SiHa (upper panel) and CaSki (lower panel) cell lines after transient over-expression of OGDHL with or without AKT1 over-expression. It is evident that OGDHL suppresses AKT activity and lead to inhibition of NF-κB-dependent gene transcription (* p<0.001). **F.** Immunoblotting analysis of phospho- NF-κB, total- NF-κB, β-actin and lamin in nuclear and cytosolic fractions of SiHa cell lines after OGDHL over-expression with or without AKT1 overexpression. The data showed that co-transfection of OGDHL and AKT1 partially increased the translocation of NF-κB from the cytoplasm to the nucleus.

A number of lines of evidence demonstrate that PI3K/AKT signaling is a major element of ROS-mediated apoptosis [10], [16], leading us to ask if this pathway is involved in OGDHL-mediated ROS production and apoptosis. To address this question, we determined the protein levels and phosphorylation state of key components of the AKT signaling cascade, using cervical cancer cell lines with either the forced expression or knock-down of OGDHL. Immunoblotting analysis showed that overexpression of OGDHL in SiHa and CaSki cell lines decreased both phospho and total AKT levels (**Fig. 5A**), though no significant change in phospho PI3K levels were observed (**Fig. 5A**). Furthermore, OGDHL down-regulation increased the phospho and total AKT levels in HeLa and ME180 cell lines (**Fig. 5B**). However, rescue experiment with OGDHL re-expression in OGDHL knockdown cells showed decrease in AKT phosphorylation compared to only OGDHL knockdown and this was comparable to control transfection (**Figure S3**). However, the mechanistic link between OGDHL and AKT need to be explored in future studies.

### OGDHL down-regulates NF-κB-transcriptional activity

Deregulation of NF-κB is a major contributor to the abnormal growth, resistance to apoptosis, and propensity to metastasize observed in many different cancers [17]. Interestingly, increased activation of NF-κB is found in many human cancers, including cervical cancer [18], [19]. Furthermore, it is now well established that AKT can activate the NF-κB signaling pathway [20], [21], [22]. After translocating into the nucleus, NF-κB can activate a variety of genes that are involved in different cellular processes including cell survival [23], [24]. Interestingly, after OGDHL overexpression in SiHa cells, NF-κB translocation from cytoplasm towards the nucleus was decreased (**Fig. 5C**). Further, OGDHL overexpression in SiHa cells decreased the DNA binding activity of NF-κB (detected by formation of a protein-DNA complex using the consensus κB binding site and nuclear extracts in mobility shift assay) compared to that of control (**Fig. 5D**). To monitor the activity of NF-κB signaling pathways, SiHa and CaSki cells were transiently transfected with a luciferase reporter plasmid driven by NF-κB response elements. Consistent with the decreased NF-κB nuclear translocation, we found that luciferase activity was also decreased in both cell lines after OGDHL co-transfection (**Fig. 5E**). Interestingly, when the cells were co-transfected with AKT1 and OGDHL, the NF-κB luciferase activity recovered significantly, while AKT1 siRNA transfection decreased the NF-κB luciferase activity in both the SiHa and CaSki cell lines, implying that NF-κB inactivation is due to AKT1 inactivation (**Fig. 5E**). To verify this observation, we determined phospho and total NF-κB protein localization after co-transfection of OGDHL and AKT1 and found that co-transfection of OGDHL and AKT1 partially increased the translocation of NF-κB from the cytoplasm to the nucleus (**Fig. 5F**). Collectively, these findings indicate that OGDHL suppresses AKT activity, followed by decreased nuclear translocation and DNA-binding activity of NF-κB, leading to inhibition of NF-κB-dependent gene transcription.

### OGDHL increases cell viability through AKT expression or Modulation of AKT levels after OGDHL overexpression

**Figure 6:**
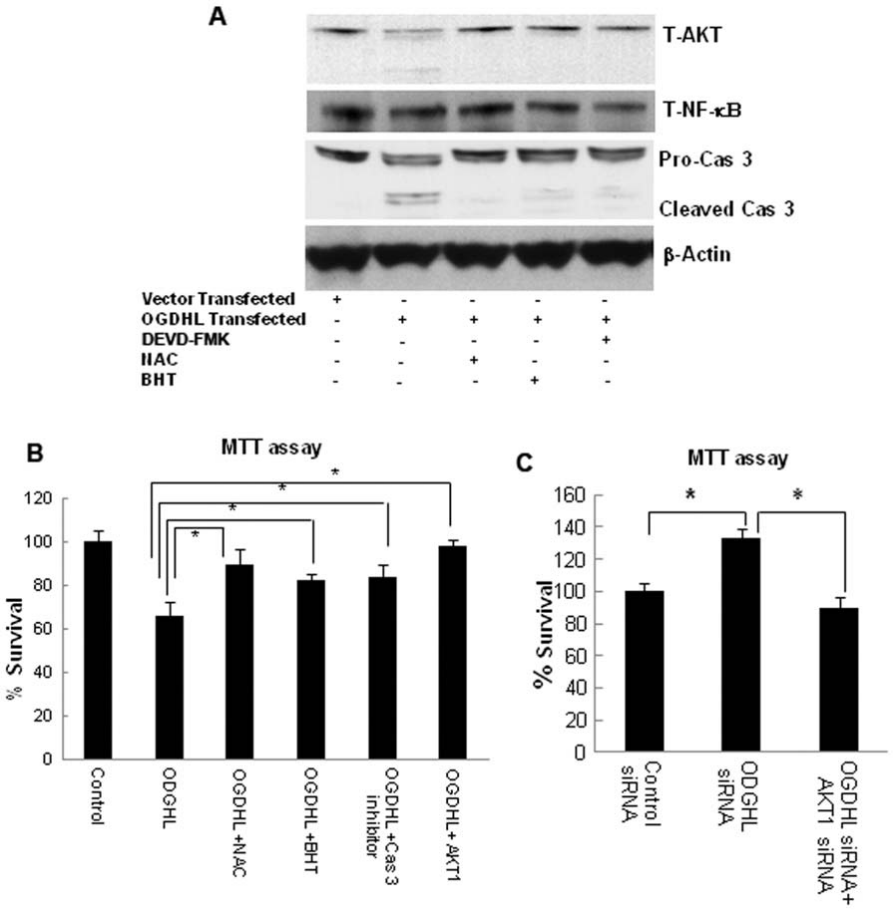
Relationship among OGDHL expression, ROS production and alterations of AKT pathway genes. **A.** Immunoblotting analysis of total-AKT (T-AKT), total NF-κB (T -NF-κB), pro and cleaved caspase 3, cleaved PARP1/2 in SiHa cell line 48 hours after OGDHL over-expression in the presence or absence of ROS (N-acetyl l-cysteine or NAC, 2.5 mM), Lipid-peroxidation (butylated hydroxytoluene or BHT, 0.2 mM) and caspase 3 (DEVD-FMK, 25 µM) inhibitor. β-actin was used as a loading control. All the inhibitors protect AKT from getting cleaved. ROS and lipid peroxidation inhibitors have protective effect on caspase 3 activation; **B.** MTT assay of SiHa cell line 48 hours after OGDHL or AKT1 over-expression in the presence or absence of NAC (ROS inhibitor), BHT (Lipid peroxidation inhibitor) or DEVD-FMK (caspase 3 inhibitor); NAC, BHT and DEVD-FMK significantly prevented cell death induced by OGDHL; **C.** MTT assay of HeLa cell line after OGDHL knockdown with or without AKT1 knockdown. AKT1 down-regulation by AKT1 siRNA decreased HeLa cell survival that was induced by knockdown of OGDHL (*P<0.05).

It was previously demonstrated that AKT is an endogenous substrate of caspase 3, and activated caspase 3 can also reduce AKT activity [25], [26]. Since we observed that OGDHL over-expression increased ROS production and lipid peroxidation with caspase 3 activation and decreased AKT protein levels in SiHa and CaSki cell lines, we examined whether AKT is cleaved by activated caspase 3. To this aim, OGDHL was over-expressed in SiHa cells in the presence or absence of caspase 3 inhibitor (DEVD-FMK), ROS inhibitor NAC, or lipid peroxidation inhibitor BHT, followed by immunoblotting (for AKT, caspase 3 and PARP1/2) and MTT assay. We found that all the inhibitors protect AKT from getting cleaved (**Fig. 6A**). Furthermore, ROS and lipid peroxidation inhibitors prohibited caspase 3 activation (**Fig. 6A**). In the MTT assay, we found that caspase 3 inhibitors, NAC and BHT, significantly prevented cell death induced by OGDHL overexpression (**Fig. 6B**). Interestingly we also found that AKT1 overexpression could also significantly diminish cell death induced by OGDHL (**Fig. 6B**), while AKT1 down-regulation by AKT1 siRNA decreased HeLa cell survival that was increased by knockdown of OGDHL (**Fig. 6C**). Furthermore, we confirmed that caspase 3 mediated AKT cleavage through *in vitro* caspase 3 assay in SiHa and CaSki cell lines by using SiHa and CaSki whole cell lysate in the presence of recombinant caspase 3 with or without caspase 3 inhibitor (DEVD-FMK) at different time points (data not shown). These cumulative data suggest that the increased oxidative stress followed by caspase 3-mediated AKT cleavage may be a mechanism by which the activation of this survival pathway is censored in cervical cancer cell lines by forced expression of OGDHL.

### OGDHL re-expression suppresses anchorage-independent growth and invasion of malignant cervical cancer cells

**Figure 7:**
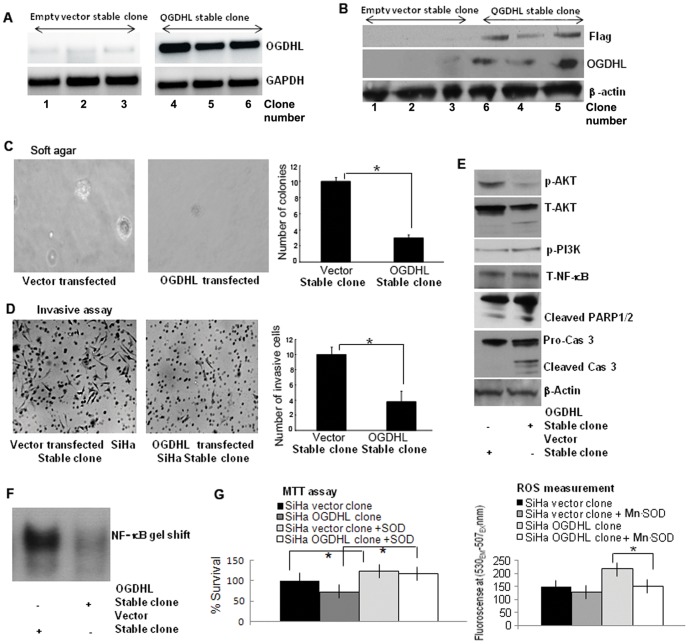
Tumor suppressive properties of OGDHL using stable clone. **A.** RT-PCR analysis of SiHa cells stably transfected with the mammalian expression vector pcDNA3 with full-length OGDHL cDNA and empty pcDNA3 vector (mock). All the three OGDHL clones (clone number at the bottom) containing full-length OGDHL cDNA expressed OGDHL at various levels. There is a very low level endogenous OGDHL expression in mock-transfected clones. **B.** Immunoblot analysis of Flag, OGDHL and β-actin. All the three OGDHL over-expressing clones showing Flag tagged with OGDHL and OGDHL expression while no Flag-OGDHL and OGDHL expression in empty vector stable clones (clone number at the bottom). **C.** Soft agar assay: number of soft agar colonies was significantly lower in the OGDHL over-expressed cells compared with empty vector-transfected cells (*P<0.001) (Bottom). Magnification, ×100 in representative photograph (Top). **D.** Invasion assay: Compared with empty vector, stable OGDHL over-expressing SiHa cell clone showed significantly less number of invading cells (*P<0.001) (right). Magnification, ×100 in representative photograph (left. Each experiment was repeated twice. In general all the findings are consistent with the transient transfection data; **E.** Immunoblotting analysis of phospho-AKT, total-AKT, phospho-PI3K, total-NF-κB and β-actin in empty vector (left lane) and OGDHL over-expressing stable SiHa cell clone (right lane) and the findings are similar to transient transfection. **F.** NF-κB gel shift assay in SiHa cell lines stably transfected with empty vector or vector containing OGDHL-Flag indicate that OGDHL inhibited the binding of NF-κB to the nuclear DNA. **G.** Inhibition of OGDHL mediated ROS generation by over-expression of Mn-SOD increases the viable cells determined by MTT assay (*P<0.05) (left panel) and this increase number of cells is partially due to decrease ROS production as shown in right panel (*P<0.05).

To investigate the significance of long term effect of OGDHL re-expression in a cervical cancer cell line, we generated OGDHL over-expressing stable clones using SiHa cell line and compared the functional and molecular differences of this cell line with the stable clones containing vector only. OGDHL stable clones were confirmed by RT-PCR for OGDHL using the cDNA obtained from this cell line (**Fig. 7A**) and by immunoblotting analysis using flag and OGDHL antibody (**Fig. 7B**). We then measured soft agar colony forming activity in comparison to the control cells transfected with an empty vector. As expected, the control SiHa cell lines readily formed colonies, indicative of their anchorage-independent growth potential (**Fig. 7C**). However, the SiHa cell line stably expressing OGDHL formed far fewer and much smaller colonies in soft agar. These data suggest that OGDHL suppresses the tumorigenicity of cervical cancer cells *in vitro*. Consistent with the transient transfection data, the invasion ability of the OGDHL over-expressing stable cell clone was greatly reduced compared to that of the empty vector transfected control SiHa cells (**Fig. 7D**).

To determine the long term downstream effects of OGDHL overexpression, we performed the same experiments as we did for the transient transfection. Immunoblotting data showed a significant decrease in total and phospho-AKT and increase in cleaved caspase 3, and cleaved PARP1/2 levels with very little difference in phospho-PI3K levels in the OGDHL stable clone compared to empty vector stable clones (**Fig. 7E**), which was consistent with the transient OGDHL overexpression data (**Fig. 3E**). Similar to the transient transfection data, a significant decrease of DNA binding activity was detected for NF-κB as measured by formation of a protein-DNA complex using the consensus κB binding site and nuclear extracts in a mobility shift assay in OGDHL stable compared to vector stable clones (**Fig. 7F**). Because transient over-expression of OGDHL increased ROS production in SiHa and CaSki cell lines, we compared ROS production and cell proliferation in both vector and OGDHL stable clones. Interestingly we found that when the cell proliferation decreased (**Fig. 7G** **, left pane**l); ROS production (**Fig. 7G** **, right panel**) was significantly increased in OGDHL stable clones compared to that of vector clones. Furthermore, when we overexpressed Mn-SOD in OGDHL stable clones, it increased the proliferation rate (**Fig. 7G** **, left panel**) and decreased the ROS production (**Fig. 7G** **, right panel**) of the control cells significantly compared to that of OGDHL stable clones. These data confirm that ROS production increases in the presence of OGDHL; OGDHL plays an important role in ROS mediated cytotoxicity.

## Discussion

In the present study, we have shown that OGDHL negatively regulates cell proliferation by inducing apoptosis in cervical cancer cells. To our knowledge, no study has demonstrated OGDHL's ability to induce apoptosis in an epithelial cell model; however, isoenzymes (eg. OGDH) of OGDHL have been shown to induce neural cell death [13], [26], [27]. Furthermore, it has been reported previously that nuclear encoded gene(s) localized in mitochondria may decrease cell proliferation and induce apoptosis in different *in vitro* and *in vivo* systems [28]. There is a high probability of the localization of this protein to mitochondria revealed by Target P (http://www.cbs.dtu.dk/services/TargetP/) [29], WoLF PSORT (http://wolfpsort.org/) [30], Predotar (http://urgi.versailles.inra.fr/predotar/predotar.html), and MitoProt (http://ihg.gsf.de/ihg/mitoprot.html) [31]. We now report for the first time the experimental evidence of OGDHL's cellular localization in this study. Our western blot analysis clearly demonstrated that OGDHL localized to mitochondria. The antibody we used for western blotting was not suitable for immunoflorosecnce study. So we used OGDHL antibody from another manufacturer for immunofluroscence study. Also we observed very faint non-specific staining in cytoplasm, co- localization of OGDHL in mitochondria with mito-tracker dye is evident.

To date, the molecular mechanisms underlying OGDHL–induced apoptosis in any *in vitro* studies have not been known. Our study showed that forced expression of OGDHL results in increased ROS production, increased caspase activation, and causes inactivation of AKT. However, an overexpression of any protein is causing effects that are not seen naturally occurring and are possibly “off target”. To confirm whether OGDHL– induced apoptosis occurs through a PI3K/AKT pathway, cells expressing high levels of OGDHL were transfected with OGDHL siRNA, and it was seen that knock-down of OGDHL completely reversed the induction of apoptosis by increasing AKT activity and decreasing caspase 3 activity. These data suggest that OGDHL– induced apoptosis occurs through a PI3K/AKT-dependent pathway. A recent study showed that OGDHL isoenzyme (OGDH) elicits production of ROS [26] and apoptosis [26]. Little is known, however, about the functional role of the AKT pathway in mediating OGDHL– induced ROS production in human cervical cancer cells. The results of the present study show that AKT inactivation is a consequence of OGDHL–mediated ROS production and makes a key functional contribution to the lethality of human cervical cancer cells. ROS are known to be a potent inducer of cytochrome *c* release mediated, caspase-dependent apoptosis [32] and our data determined that forced expression of OGDHL increased Cytochrome C release in SiHa cells (**Figure S1C**).

AKT is activated in response to many growth factors [33]. It inhibits apoptosis through multiple pathways (40) that induce direct phosphorylation and inactivation of pro-apoptotic proteins, such as BCL2-antagonist of cell death, or procaspase-9; increased expression of c-IAPs, c-FLIPL, Bcl-2, Mcl-1; and down-regulation of pro-apoptotic proteins [33], [34]. Activation of AKT occurs in response to oxidative damage [20], which generally involves PTEN inactivation and results in an attenuation of lethality. The present findings suggest that the relationship between OGDHL–mediated ROS production and the effects on AKT activity differ from those of a previous report showing that ROS activate AKT [20]. Most notably, in this study OGDHL over-expression resulted in diminished, rather than increased, AKT phosphorylation. This current finding is consistent with that of a previous study showing that 15d-PGJ2 induces down-regulation of phospho-AKT and apoptosis in leukemia [35].

ROS play critical roles in the regulation of proliferation, apoptosis, and cellular transformation [20], [36], [37]. It has recently been reported that KDGHC (an isoenzyme of OGDHL) is a primary resource of ROS production in mitochondria [26]; however, in our study the mechanism of induction of ROS by OGDHL remains unknown. In this study, we used siRNA mediated knockdown of OGDHL to investigate the involvement of ROS in OGDHL–mediated apoptosis and showed that mitochondria and NADPH oxidase are involved in ROS production by OGDHL. Several lines of evidence suggest that ROS play an essential role in OGDHL–mediated apoptosis in cervical cancer cells. SiRNA knockdown of OGDHL leads to decreased ROS production in two cervical cancer cell lines (**Fig. 2A** **right panel**) and significantly inhibits OGDHL–induced cell death (**Figure S2**). In addition, OGDHL knock down blocks PARP cleavage resulting from OGDHL expression and prevents inactivation of AKT and the down-regulation of AKT mRNA and protein levels. Together, these data indicate that AKT activity represents a critical signaling node operating downstream of OGDHL–induced oxidative stress, which triggers mitochondrial injury and engagement of the apoptotic caspase cascade. Future studies will be aimed at defining the mechanisms by which ROS lead to a down regulation of AKT.

Previous studies suggested that ROS production leads to activation of extracellular signal-regulated kinase 1/2 (ERK1/2) and c-Jun-N-terminal kinase (JNK) and then activation of the NF-κB transcription factor [38]. To investigate the role of ERK activation in OGDHL–induced apoptosis, we used western blot analysis to examine the expression of p-ERK after transfection with OGDHL. The intensity of activation was not significantly different between OGDHL stable cell lines and the empty vector transfectants (data not shown). Thus, the decrease in anchorage-independent growth and cell proliferative properties of the OGDHL stable cell lines are not because of the inactivation of the ERK signal cascade, but are properties of induction of other pro-apoptotic signals like AKT and NF-κB, determined in the present study. Further studies are underway to understand the detailed molecular relationship between these two molecules and which additional signaling pathways are affected by their interaction.

NF-κB is a ubiquitous transcription factor that is activated by a variety of proinflammatory cytokines, bacterial endotoxins, viral infection, DNA damage, and free radicals [39]. In unstimulated cells under normal physiological conditions, NF-κB is maintained in the cytoplasm as an inactive complex with members of the IκB inhibitor family [23], [40], [41], [42], [43] . This interaction inhibits nuclear translocation and the DNA binding activity of NF-κB. Cellular stimulation leads to phosphorylation, ubiquitination, and subsequent proteolysis of IκB in proteasomes, enabling NF-κB to translocate into the nucleus and to activate a variety of genes including cytokines, cell cycle-regulatory genes, and anti-apoptotic genes. Constitutive NF-κB activation is frequently associated with a number of pathological conditions, including inflammation and cancer and is well known to be involved in tumor angiogenesis and invasiveness [44]. Aberrant activation of NF-κB is frequently observed in many cancers. Moreover, suppression of NF-κB limits the proliferation of cancer cells. Loss of the normal regulation of NF-κB is a major contributor to the deregulated growth, resistance to apoptosis, and propensity to metastasize observed in many different cancers [17]. OGDHL directly binds to NF-κB, and over-expression of OGDHL significantly diminishes the transcriptional activity of NF-κB. Hence, OGDHL has potential therapeutic applications in cervical cancer through inhibition of NF-κB signaling.

Protein kinase B/AKT kinase, a serine/threonine kinase, is the core component of the phosphoinositide 3-kinase/AKT signaling pathway, which is one of the most frequently hyperactivated signaling pathways in human cancers [45], [46], [47]. AKT was shown to regulate many of the key effector molecules involved in apoptosis and cell cycle arrest. Once activated, AKT can translocate from the cellular-membrane into the cytoplasm and nucleus and phosphorylate numerous proteins, leading to tumor development or progression, as well as resistance to treatment with chemotherapy and/or radiation therapy [48]. Additionally, it is known that AKT can activate NF-κB signaling pathway [20], [21], [22]. Previous reports demonstrated that AKT is an endogenous substrate of caspase 3 and that activated caspase 3 can also reduce AKT activity [25], [26].

Interestingly, our findings show that over-expression of OGDHL in cervical cancer cell lines blocks AKT activation and inhibit cervical cancer cell proliferation and survival. Furthermore, decreased AKT activity, cell proliferation, and survival changes are significantly blunted in cells transfected by siRNA–mediated OGDHL knockdown. Taken together, our findings indicate that down-regulation of OGDHL promotes cervical cancer cell proliferation and survival. We and others reported previously that OGDHL is inactivated due to promoter methylation and cancer specific promoter methylation occurs in several different cancer types [1], [5], [6]. Aberrant methylation of genes can occur as a consequence of, or in association with, signaling of major pathways activated by genetic alterations. For example, methylation of T lymphocyte genes in lupus [49], the tumor suppressor gene *Par-4* in transformed epithelial cells [50] and *p16(INK4A*), *p21(WAF1*) [51] and *hMLH1* [52] genes in colon cancer cells was found to be closely linked to the MAP kinase pathway with *BRAF* mutation. Here we have shown that methylation in SiHa cell leads to suppression of OGDHL, and it may be the first example of epigenetic alteration of OGDHL linked to the PI3K/AKT pathway. We have tested the PI3K/AKT signaling in cervical cancer cell lines with different baseline levels of OGDHL expression, which may not necessarily reflect the behavior of primary tumors. However we found about 20% incidence of OGDHL methylation in cervical cancer, and we thus presume that at least some cervical cancers may develop due to inactivation of the *OGDHL* gene by promoter methylation and activation of the AKT- NF-κB pathway. However, it remains to be seen whether methylation of the *OGDHL* gene can result in the aberrantly activated signaling of the PI3K/AKT pathway. The level of natural *OGDHL* methylation was generally low in the cervical cancer cell lines tested in the present study. Therefore, more appropriate cell models may be needed to study the relationship between *OGDHL* methylation and the aberrant PI3K/AKT signaling. Cancer is a complex and multifactorial disease and any single molecule or pathway may not involve in every primary cervical cancer. In depth molecular studies for any gene (here OGDHL) that altered in cervical cancer will help to develop personalized therapy in future.

In summary, our study shows that OGDHL induces apoptosis of cervical cancer cells via an AKT-dependent pathway. The effect includes generation of ROS through mitochondria and NADPH oxidase activation, negative regulation of AKT transcription levels, and inactivation of the AKT protein followed by inactivation of the NF-κB signaling pathway. The anti-apoptotic, oncogenic PKB/AKT kinase pathway is widely implicated in the origin and/or progression of human tumors (3–6). AKT was shown to regulate many of the key effector molecules involved in apoptosis and cell cycle arrest [45]. Although the relevance of AKT signaling in cancer and chemoresistance is widely accepted, the exact mechanism of AKT regulation in human cancer cells remains incomplete. Here, we showed that OGDHL modulates AKT signaling in cervical cancer cell lines, and thus our results suggest that OGDHL may have biologic importance in initiation and progression of cervical cancer and it may be use as a biomarker for the management of cervical cancer.

## Supporting Information

Figure S1 Expression and localization of OGDHL after transient transfection: **A.** mRNA (upper panel) and protein (lower panel) expression of OGDHLdetermined by semi-quantitative RT-PCR and immunoblotting analysis respectively in SiHa and CaSki cell lines after 48 hours of forceful expression of OGDHL and empty vector transfection; **B.** mRNA and protein expression of OGDHL in HeLa and ME180 cell lines determined by semi-quantitative RT-PCR (top panel) and immunoblot (lower panel) analysis 48 hours after transient transfection of OGDHL siRNA and scramble siRNA (control siRNA) as indicated in the methods; **C.** Nuclear, mitochondria and cytosolic fractions of SiHa cell lines 48 hours after OGDHL-flag overexpression. β-actin: control for cytosolic protein, lamin: Control for nuclear protein and Cox-II : Control for mitochondrial protein; Cytochrome C release increased in SiHa cells after OGDHL overexpression.
(TIF)

Figure S2 Rescue of OGDHL in HeLa and ME180 cells after SiRNA mediated downregulation. OGDHL was rescued by forceful expression 16–24 hours after OGDHL siRNA transfection. A: MTT assay 48 hours after transient transfection of OGDHL siRNA, controls and OGDHL rescued HeLa and ME180 cell lines; Cell growth rate is expressed as absorbance at 570 to 650 nm ( *P<0.001). B. Caspase 3 assay: Caspase-3 activities in HeLa and ME180 cells were measured in scramble or OGDHL siRNA-transfected cells and OGDHL rescued cells after 48 hours. (*P<0.001 in each comparison). C. Immunoblotting analysis of cleaved Caspase 3 and PARP1/2 in HeLa and ME180 cell lines 48 hours after transfection . and D. Invasive assay 48 hours after transient downregulation of OGDHL, controls and OGDHL rescued HeLa cells as described in the methods. HeLa cells that invaded the polycarbonate membrane of transwell chamber (Left). E. The number of HeLa cells that invaded the polycarbonate membrane of transwell chamber (*P<0.001). The data represent the mean ±SD of three independent experiments, each done in triplicate.
(TIF)

Figure S3 Immunoblotting analysis of phospo-AKT, total-AKT and β-actin in HeLa and ME180 cell lines 48 hours after transfection of scramble siRNA for OGDHL, OGDHL siRNA-and cotransfection of OGDHL siRNA and OGDHL overexpression construct. The data clearly indicated that cotransfection of OGDHL siRNA and OGDHL overexpressing vector decrease the p-AKT almost similar to control OGDHL siRNA.
(TIF)
